# Scribner’s Monthly

**Published:** 1873-08

**Authors:** 


SCRIBNER'S MONTHLY commenced in August a thrilling tale from the pen of Bret Harte, entitled, "An Episode of Fiddletown," for which the publishers paid one thousand dollars. In November will be commenced "Annals of an English Abbey," by Mr. Fronde, for which they also paid one thousand dollars. R. H. Stoddard is preparing a series of splendid articles, while Ed. King will, in the September number, give "The Paris of America," the second of the Great South Series. Dr. J. G. Holland—who, by the way, is an M.D., and one who, the profession should feel, has "stooped to conquer" in literary pursuits—is the conductor of this monthly. If he is as skillful and scientific a physician as he is a writer and editor, he is certainly among the M.D. peerage of the land. His "Arthur Bonnicastle" is a gem of the first water. Send for *Scribner*.

